# To and TAFRO – a cryptic cause of acute renal failure: a case report

**DOI:** 10.1186/s12882-022-02660-7

**Published:** 2022-01-06

**Authors:** N. Shah, T. Davidson, C. Cheung, K. Keung

**Affiliations:** 1 Department of Nephrology, Prince of Wales Hospital, Randwick, New South Wales Australia; 2 Faculty of Medicine, University of New South Wales, Kensington, New South Wales Australia; 3 New South Wales Health Pathology East, Prince of Wales Hospital, Randwick, New South Wales Australia; 4 Department of Haematology, Prince of Wales Hospital, Randwick, New South Wales Australia

**Keywords:** Acute renal failure, Kidney biopsy, Case report

## Abstract

**Background:**

TAFRO syndrome is a rare clinical subtype of idiopathic multicentric Castlemans disease characterised by thrombocytopenia, anasarca, myelofibrosis, renal dysfunction, and organomegaly. Renal involvement is common, sometimes requiring temporary renal replacement therapy. Due to the associated thrombocytopenia, renal biopsies are rarely performed limiting descriptions of the renal histopathology in this condition. This case describes a patient with TAFRO syndrome and the associated renal histology.

**Case presentation:**

A 49-year-old Caucasian man presented to a tertiary hospital in Sydney with a six- week history of malaise, non-bloody diarrhoea, progressive shortness of breath, and drenching night sweats. A progressive bicytopenia and renal function decline necessitating temporary dialysis prompted a bone marrow aspirate and trephine, as well as a renal biopsy respectively. This noted a hypercellular bone marrow with increased granulopoiesis, reduced erythropoiesis, and fibrosis, with renal histology suggesting a thrombotic microangiopathic-like glomerulopathy. Alternate conditions were excluded, and a diagnosis of TAFRO syndrome was made. Glucocorticoids and rituximab were initiated with rapid renal recovery, and normalisation of his haematologic parameters achieved at six months.

**Conclusion:**

This case describes an atypical thrombotic microangiopathy as the predominant histologic renal lesion in a patient with TAFRO syndrome. This was responsive to immunosuppression with glucocorticoids and rituximab, highlighting the importance of early recognition of this rarely described condition.

## Background

TAFRO syndrome is a rare systemic inflammatory disease characterised by a constellation of symptoms including thrombocytopenia, anasarca, myelofibrosis, renal dysfunction, and organomegaly. Initially described in Japanese patients in 2010, TAFRO is now recognised as a subclass of Human herpesvirus-8 (HHV-8)-negative, idiopathic multicentric Castleman disease (iMCD) [1, 2]. A diagnosis of TAFRO requires the presence of 3 major criteria (anasarca, platelet count < 100, and systemic inflammation defined as a temperature > 37.5^o^ Celsius or a C-reactive protein of > = 0.2 mg/L), 2 of 4 minor criteria (Castleman disease-like features on a lymph node biopsy, reticulin myelofibrosis and/or increased megakaryocytes on bone marrow biopsy, mild organomegaly including hepatomegaly/splenomegaly/enlarged lymph nodes, and progressive renal dysfunction) without any of the exclusion criteria (malignancies, autoimmune disorders, infectious disorders, POEMS syndrome, hepatic cirrhosis, and TTP/HUS) [3]. Although renal dysfunction is seen in about 55% of patients [3], the concurrent thrombocytopenia has commonly served as a relative contraindication to renal biopsy which has limited histologic descriptions of renal pathology in this condition. To our knowledge, there are less than 25 cases of TAFRO with accompanying renal histology which have noted predominantly membranoproliferative glomerulonephritis (MPGN)-like lesions or thrombotic microangiopathy (TMA)-like glomerulopathy [4]. To our knowledge, this is the first case of TAFRO syndrome described in Australia in a Caucasian man noting a TMA-like glomerulopathy on renal biopsy that was successfully treated with glucocorticoids and rituximab therapy.

## Case presentation

A 49-year-old Caucasian man presented to the Emergency Department at a tertiary hospital in Sydney with a 3-week history of generalised malaise, non-bloody diarrhoea, abdominal bloating, shortness of breath on exertion, lower limb pitting oedema, and drenching night sweats. There was no significant past medical history, with no regular medication use. Admission vital signs included a temperature of 37.8 degrees Celsius, a sinus tachycardia with a heart rate of 108 beats per minute, a normal blood pressure of 122/86 mmHg and a respiratory rate of 18 breaths per minute. Physical examination revealed a petechial rash on both lower limbs along with significant pitting oedema to the knees. Abdominal distention and shifting dullness was present without appreciable hepatosplenomegaly. There was no cervical, axillary, or inguinal lymphadenopathy on examination. On laboratory testing, the serum urea was 14.5 mmol/L, creatinine 221umol/L, potassium 6.0 mmol/L, ALP 178 U/L, haemoglobin 102 g/L, platelets 78 X 10^9/L, ESR 75 mm/h, CRP 105 mg/L, INR 1.4, fibrinogen 6.9 g/L, and D-dimer 17.19 mg/L. Urinalysis was bland, with no significant proteinuria (albumin/creatinine ratio 1.1 mg/mmol), or microscopic haematuria (RBC < 10 X 10^6^/L). Hyaline (48,180 X 10^3^/L) and granular (494 X 10^3^/L) casts were noted on microscopy. A chest x-ray showed bilateral pleural effusions, with otherwise clear lung fields. A non-contrast CT scan of the kidneys, ureters, and bladder showed small volume abdominal ascites with mild splenomegaly (measuring at 15 cm) without abdominal lymphadenopathy or renal tract abnormalities. Stool culture failed to identify an infective cause of diarrhoea.

**Fig. 1:**
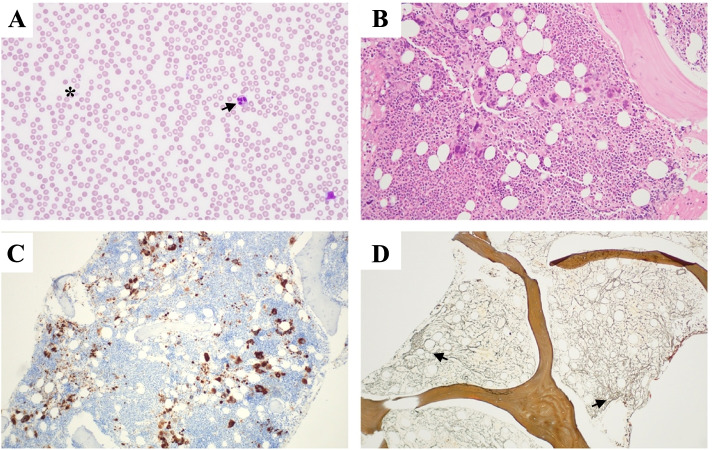
Hematologic Investigations: **A**) peripheral blood film – mild anisocytosis, hypersegmented neutrophils (black arrow), occasional giant platelets (asterisk) **B**) Bone Marrow Trephine (H&E stain) – hypercellular with normal lamellar bone **C**) Bone Marrow Trephine (CD61) – Megakaryocyte clustering and dysplasia. **D**) Bone Marrow Trephine (Reticulin) – consistent with grade 2 fibrosis (black arrows)

**Fig. 2:**
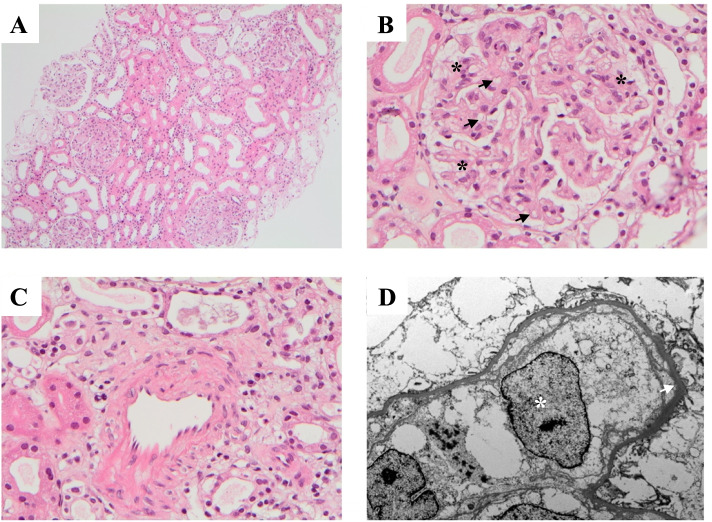
Renal Biopsy: **A**) Tubulointerstitium (H&E stain) - no significant interstitial fibrosis, tubular atrophy, or interstitial inflammation. **B**) Glomerulus (H&E stain) - mild expansion of mesangial matrix with patch endocapillary cellularity (black asterisks) and focal double contours (black arrows). No thrombi, sclerosing lesions, or necrotising lesions. **C**) Artery (H&E stain) – normal thickness with no fibrointimal hyperplasia or hylanosis. No evidence of arteritis, vasculitis or cholesterol emobli. **D**) Electron micrograph – swollen mesangial cells (white asterisks) with no electron-dense deposits or abnormal fibrils. Diffuse glomerular basement membrane thickening (white arrow). Tubules, interstitium, and vessels predominantly normal

A peripheral blood film noted microcytic and hypochromic red blood cells without the presence of teardrop poikilocytes, schistocytes, blasts, or other morphologically diagnostic or sinister findings (Fig. 1). No monoclonal paraprotein was found, Parvovirus-B19 serology was negative, and haemoglobin electrophoresis did not suggest the presence of a hemoglobinopathy. Hepatitis B, Hepatitis C, and HIV serology were negative. Aside from a low-titre anti-nuclear antigen antibodies (ANA) of 1:80, serum autoantibodies including antineutrophil cytoplasmic antibodies (ANCA), extractable nuclear antigen antibodies (ENA), and rheumatoid factor were all negative. His progressive, and symptomatic, bicytopenia (haemoglobin 85 g/L, platelets 38 X 10^9/L) requiring intermittent transfusions of packed red blood cells prompted a bone marrow aspirate and trephine. This identified a hypercellular marrow with increased granulopoiesis, reduced erythropoiesis, megakaryocyte clustering and dysplasia in association with grade 2 fibrosis (Fig. 1). A subsequent JAK2 V617F, CALR, and MPL mutation analysis was negative. With the renal function deteriorating, a kidney biopsy was performed with simultaneous platelet transfusion. Light microscopy showed mild expansion of the mesangial matrix, with increased endocapillary cellularity of the glomeruli (Fig. 2B), with variable thickening of the capillary walls and patchy double contour changes. There were no thrombi, sclerosing or necrotising lesions. Additionally, there was no tubular atrophy, interstitial fibrosis, or interstitial inflammation, and no features suggestive of an arteritis, vasculitis, or cholesterol emboli (Fig. 2). Congo red staining was negative. Immunofluorescence and C4d staining were unremarkable. The overall appearance of the biopsy was in keeping with an atypical thrombotic microangiopathy. The electron microscopy had significant reprocessing artefact but excluded immune-complex deposition, and demonstrated an expanded mesangium occupied by swollen cells and diffuse glomerular basement membrane (GBM) thickening. (Fig. 2D). Oligo-anuria with worsening pulmonary oedema prompted the initiation of haemodialysis at a creatinine peak of 531 μmol/L. A whole-body PET scan noted low metabolically active nodal disease above and below the diaphragm (SUV_max_ 4.2), diffuse metabolic activity throughout the bone marrow and spleen, as well as pleural effusions, a pericardial effusion and abdominal ascites. A decision was made to forego a lymph node biopsy as the patient already fulfilled criteria for a diagnosis of TAFRO syndrome (meeting all 3 of the major criteria, and 3 of the 4 minor criteria), having excluded other possible haematologic and autoimmune conditions, 2 weeks into his hospital admission. Pulse intravenous methylprednisolone was administered over 3 consecutive days, followed by daily oral prednisolone therapy. Although the HHV8 PCR was negative, the initial Interleukin-6 (IL-6) sample was not collected in the appropriate manner with the repeat sample being sent 1 week after initiation of methylprednisolone. This made the result (< 2.1 pg/mL) difficult to interpret. The renal dysfunction improved rapidly upon steroid initiation, with only 4 sessions of dialysis required during the admission. After 25 days in hospital, and 9 doses of glucocorticoid, the creatinine had improved to 165 μmol/L, with a persistent bicytopenia (Hb 105 g/L, Plts 51 X 10^9/L). After discharge, the patient received 800 mg of intravenous rituximab (375 mg/m^2^) administered weekly over 4 weeks, with the prednisolone weaned gradually. Over 6 months of follow-up, the bicytopenia resolved (Hb 135 g/L, Plts 246 X 10^9/L) and the renal function stabilised with a creatinine of 100–110 μmol/L, eGFR 70–75 mL/Min/1.73m^2^, without proteinuria. Repeat testing of VEGF and IL-6 10 months from the initial presentation confirmed elevated levels of 1155 ng/L, and 410.0 ng/L, respectively. Given these results, an IL-6 inhibitor, siltuximab, was initiated.

## Discussion and conclusions

This case represents a classic presentation of TAFRO syndrome, which remains an under-recognised condition, particularly in the nephrology field. There are no known biomarkers that are specific for diagnosing TAFRO making the diagnosis one of exclusion. Despite more than 50% of TAFRO cases describing renal dysfunction as a principal feature, less than 25 cases in the literature have reported the renal histology associated with this disease [4]. Thrombocytopenia has been reported in up to 100% of patients with TAFRO syndrome [5], challenging the safety of performing a renal biopsy. As summarised in a recent literature review, previous cases of renal pathology have reported thrombotic microangiopathy (TMA)-like glomerulopathy and mesangioproiferative-like glomerulonephritis [4]. Similar to these studies this case found TMA-like changes with no evidence of disease chronicity.

Treatment with glucocorticoids and rituximab was successful at inducing remission in this case. Albeit not part of the proposed diagnostic criteria, elevated levels of IL-6 and VEGF are known to be a feature of TAFRO syndrome, have been proposed as evidence supporting the diagnosis, and are considered in treatment decisions [6]. IL-6 is a soluble cytokine with pleiotropic effects across a vast number of biological processes including organ development, acute-phase reactions, inflammation, and cellular immunity, whilst VEGF promotes cell survival, angiogenesis, and vascular permeability [7]. Glucocorticoids are commonly used in the treatment of patients with severe disease, however when used as a single agent relapses are common [8, 9]. IL-6 inhibitors, such as siltuximab and tocilizumab, have the best evidence but neither are licenced by the Therapeutic Goods Administration (TGA) for use in Australia for this indication making treatment with these agents strictly off-label [6, 10]. Other therapies have been adopted from the treatment of standard forms of multicentric Castleman’s disease with case reports showing selective proteasome inhibitors, and anti-CD-20 treatments such as Rituximab capable of inducing disease remission in patients with TAFRO syndrome [11, 12]. Traditional chemotherapeutic agents are generally avoided due to their increased toxicity, and frequent relapses. As the exact pathogenesis of TAFRO is not fully understood, the most appropriate targeted therapy remains unclear. As more research is done looking for a unifying model of the underlying pathogenesis, a more detailed understanding of this condition will shed light into the efficacy of various treatment regimens.

The importance of this case lies in its ability to add to the otherwise limited description of renal histology in patients with TAFRO syndrome. In this case, an atypical TMA-like glomerulopathy was noted on renal biopsy. Treatment with immunosuppression, as demonstrated by this case, can be highly effective in inducing disease remission highlighting the importance of early recognition and treatment.

## Data Availability

Data sharing is not applicable to this article as no datasets were generated or analysed during the current study.

## Abbreviations

ANA: Anti-nuclear antigen antibodies
ANCA: Antineutrophil cytoplasmic antibodies
eGFR: Estimated glomerular filtration rate
ENA: Extractable nuclear antigen antibodies
GBM: Glomerular basement membrane
Hb: Haemoglobin
HHV8: Human herpesvirus-8
IL-6: Interleukin-6
iMCD: Idiopathic multicentric Castleman disease
MPGN: Membranoproliferative glomerulonephritis
Plts: Platelets
TGA: Therapeutic Goods Administration
TMA: Thrombotic microangiopathy
VEGF: Vascular endothelial growth factor
